# Impaired Wound Healing of Alveolar Lung Epithelial Cells in a Breathing Lung-On-A-Chip

**DOI:** 10.3389/fbioe.2019.00003

**Published:** 2019-01-22

**Authors:** Marcel Felder, Bettina Trueeb, Andreas Oliver Stucki, Sarah Borcard, Janick Daniel Stucki, Bruno Schnyder, Thomas Geiser, Olivier Thierry Guenat

**Affiliations:** ^1^ARTORG Center, Medical Faculty, University of Bern, Bern, Switzerland; ^2^HES-SO, Institute of Life Technologies, Sion, Switzerland; ^3^AlveoliX, Bern, Switzerland; ^4^Pulmonary Medicine Department, University Hospital of Bern, Bern, Switzerland; ^5^Thoracic Surgery Department, University Hospital of Bern, Bern, Switzerland

**Keywords:** wound healing, organ-on-a-chip, air–blood barrier, cyclic stretch, idiopathic pulmonary fibrosis

## Abstract

The lung alveolar region experiences remodeling during several acute and chronic lung diseases, as for instance idiopathic pulmonary fibrosis (IPF), a fatal disease, whose onset is correlated with repetitive microinjuries to the lung alveolar epithelium and abnormal alveolar wound repair. Although a high degree of mechanical stress (>20% linear strain) is thought to potentially induce IPF, the effect of lower, physiological levels of strain (5–12% linear strain) on IPF pathophysiology remains unknown. In this study, we examined the influence of mechanical strain on alveolar epithelial wound healing. For this purpose, we adopted the “organ-on-a-chip” approach, which provides the possibility of reproducing unique aspects of the *in vivo* cellular microenvironment, in particular its dynamic nature. Our results provide the first demonstration that a wound healing assay can be performed on a breathing lung-on-a-chip equipped with an ultra-thin elastic membrane. We cultured lung alveolar epithelial cells to confluence, the cells were starved for 24 h, and then wounded by scratching with a standard micropipette tip. Wound healing was assessed after 24 h under different concentrations of recombinant human hepatic growth factor (rhHGF) and the application of cyclic mechanical stretch. Physiological cyclic mechanical stretch (10% linear strain, 0.2 Hz) significantly impaired the alveolar epithelial wound healing process relative to culture in static conditions. This impairment could be partially ameliorated by administration of rhHGF. This proof-of-concept study provides a way to study of more complex interactions, such as a co-culture with fibroblasts, endothelial cells, or immune cells, as well as the study of wound healing at an air–liquid interface.

## Introduction

An organ-on-a-chip (OOC) is an advanced *in vitro* system that provides cells with an environment that closely resembles their *in vivo* milieu (Benam et al., 2015). In such environments, cells maintain their original functions and respond to external stimuli, such as mechanical forces or therapeutic compounds, as if they were within the tissues of the body. OOCs also recapitulate aspects of healthy and diseased cellular environments, allowing investigation of fundamental biological questions. In contrast to *in vivo* studies, OOCs make it possible to individually assess the effects of specific parameters of the microenvironment. Thus, OOCs are promising tools for improving and accelerating drug development, as well as obtaining deeper insights into pathophysiological processes (Chan et al., 2013; Sackmann et al., 2014; Esch et al., 2015).

Idiopathic pulmonary fibrosis (IPF) is a chronic, fatal lung disease characterized by an abnormal wound healing and remodeling process of alveolar epithelial cells (King et al., 2011). The various aspects of the disease and the lack of predictive *in vivo* and *in vitro* models make IPF difficult to study. It is generally accepted that IPF is initiated by repetitive microinjuries to the alveolar epithelium. Aberrant repair of those injuries is followed by a fibro-proliferative response that induces progressive scarring of the lung parenchyma, ultimately leading to respiratory failure. Although the cause of these microinjuries, as well as the cause of aberrant wound healing, remains unknown, it is thought that mechanical forces play important roles in the development and progression of IPF (Selman and Pardo, 2014). In particular, scarring of the lungs occurs with higher probability at the base and periphery, where mechanical forces due to breathing are greatest. Under physiologic conditions, alveolar regions of the lungs are exposed to a linear strain of up to 12% (Waters et al., 2012). However, scarred areas are less elastic than the healthy elastic regions and must compensate for the strain, resulting in higher mechanical stretch in those areas. High mechanical stretch causes apoptosis and slows wound repair in rat alveolar epithelial cells and decreases cell migration in bronchial 16HBE-14o-cells (Desai et al., 2008; Crosby et al., 2011). In addition, wound repair of primary human alveolar epithelial cells is delayed under physiologic (10%) linear strain (Ito et al., 2014). Thus far, the *in vitro* systems used to investigate wound healing have been based on stiff, Petri dish–based systems or stretchable systems assembled on thick silicone substrates (Guenat and Berthiaume, 2018). We recently demonstrated that microfluidics platforms enable investigation of alveolar epithelial microinjuries more reproducibly and at a smaller scale (Felder et al., 2012, 2014). The hypothesis that gastro-esophageal reflux is a source of repetitive microinjuries was also investigated on a chip (Felder et al., 2014).

Here, we report the use of a microengineered lung-on-a-chip (Stucki et al., 2015, 2018) with a “breathing” ultra-thin membrane to investigate the effects of cyclic mechanical stress induced by respiratory motions on alveolar wound repair. The open design of the lung-on-a-chip enabled the use of a scratch assay to create microinjuries in the intact alveolar epithelium. The epithelium was further exposed to hepatic growth factor, which promotes alveolar epithelial wound healing (Felder et al., 2012). The results confirmed that physiological mechanical stress affects alveolar wound closure. These results are a first step toward recapitulating the complex interplay of wound repair in the alveolar epithelium.

## Methods

### Fabrication of the Lung-On-A-Chip

**Figure 1:**
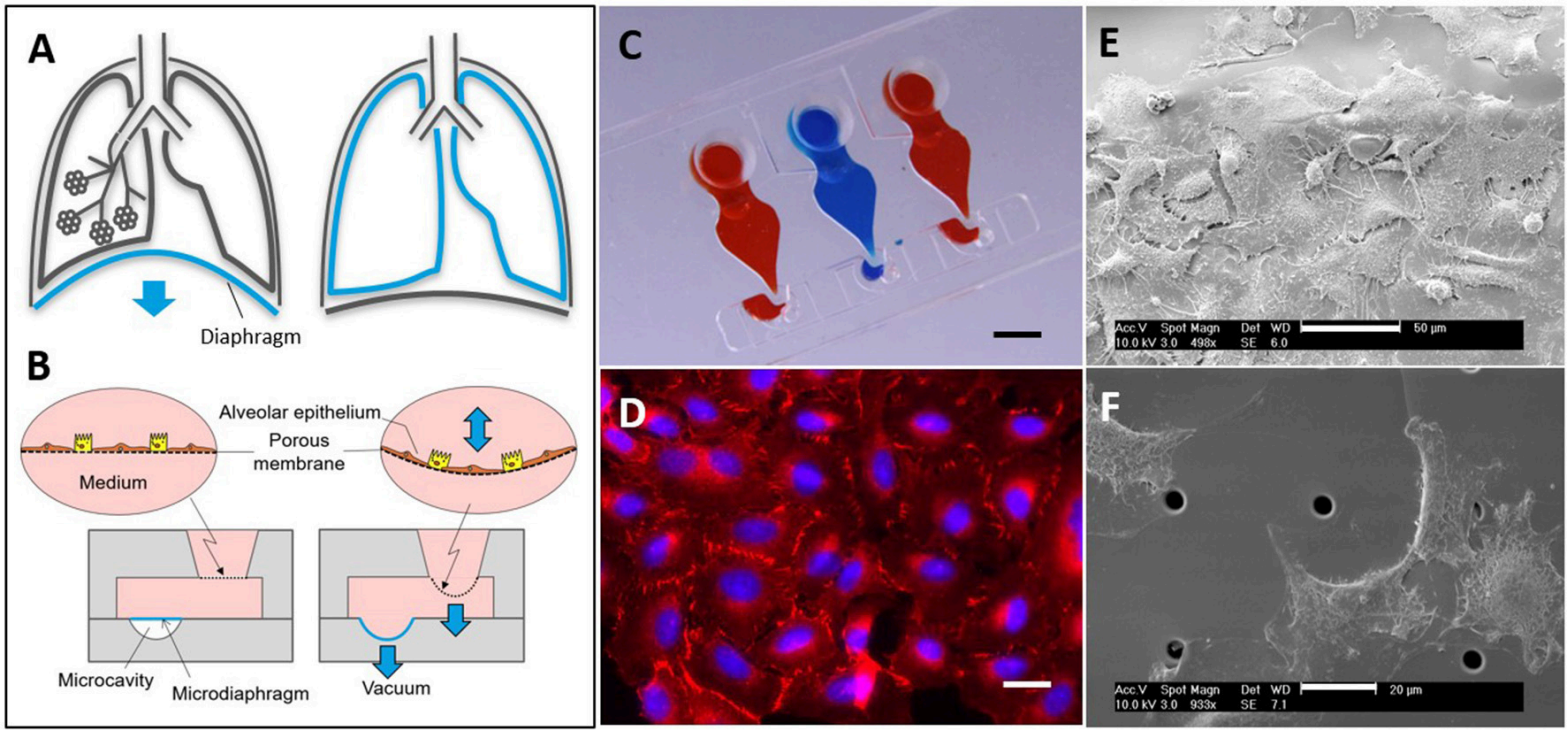
**(A)** Breathing concept *in vivo*. When the diaphragm contracts, negative thoracic pressure is generated and the lungs and lung alveoli expand. **(B)** Breathing concept *in vitro*. A microdiaphragm deflects in a defined microcavity. Due to the negative pressure generated, the alveolar membrane, where the cells are cultured, deflects downward. Schematic adapted from (Guenat and Berthiaume, 2018). **(C)** Image of the lung-on-a-chip with three culture chambers. Scale bar: 3 mm. **(D)** Immunofluorescence image of A549 cells cultured on the membrane. Cell nuclei are stained in blue, and ZO-1 (a marker of tight junctions) is stained in red. Scale bar: 20 μm. **(E,F)** Scanning electron micrograph of A549 on non-porous (top) and porous (bottom) membranes. Scale bars: 50 μm (top), 20 μm (bottom).

The working principle and fabrication of the lung-on-a-chip were described previously (Stucki et al., 2015, 2018). Briefly, the bioinspired lung-on-a-chip consists of an ultra-thin alveolar membrane that is deflected by the action of a microdiaphragm to mimic the mechanical strain of breathing (Figures 1A–C). The alveolar membrane, on which cells can be cultured on both sides, consists of a 3.5 μm–thick elastic membrane (with or without 3 μm pores, 800,000 pores/cm^2^) made of polydimethylsiloxane (PDMS, Sylgard 184, Dow Corning). The membrane is sandwiched between an apical and a basal cell culture chamber. The microdiaphragm is a 40 μm–thick membrane located at the bottom of the basal chamber and actuated by an external electro-pneumatic system.

### Cell Culture

A549 lung alveolar epithelial-like cells (ATCC cat#: CCL-185, RRID:CVCL_0023) were cultured on fibronectin-coated PDMS membranes (2.5 μg/cm^2^, Corning) in RPMI 1640 culture medium with GlutaMAX™ (Gibco/Life Technologies, cat#: 61870-010) supplemented with 10% fetal bovine serum (FBS, Gibco/Life Technologies, cat#: 10270-1 06) and 1% penicillin–streptomycin (Gibco Life Technologies, cat#: 15140-122) at 37°C and 5% CO_2_. The cells were seeded at 50,000 cells/cm^2^ on the apical side of the alveolar membrane. Cell culture medium was exchanged daily. When the cells reached confluence after 48 h, they were cultured in starvation medium (RPMI 1640 without FBS) for an additional 24 h.

### Wound-Healing Assay

**Figure 2:**
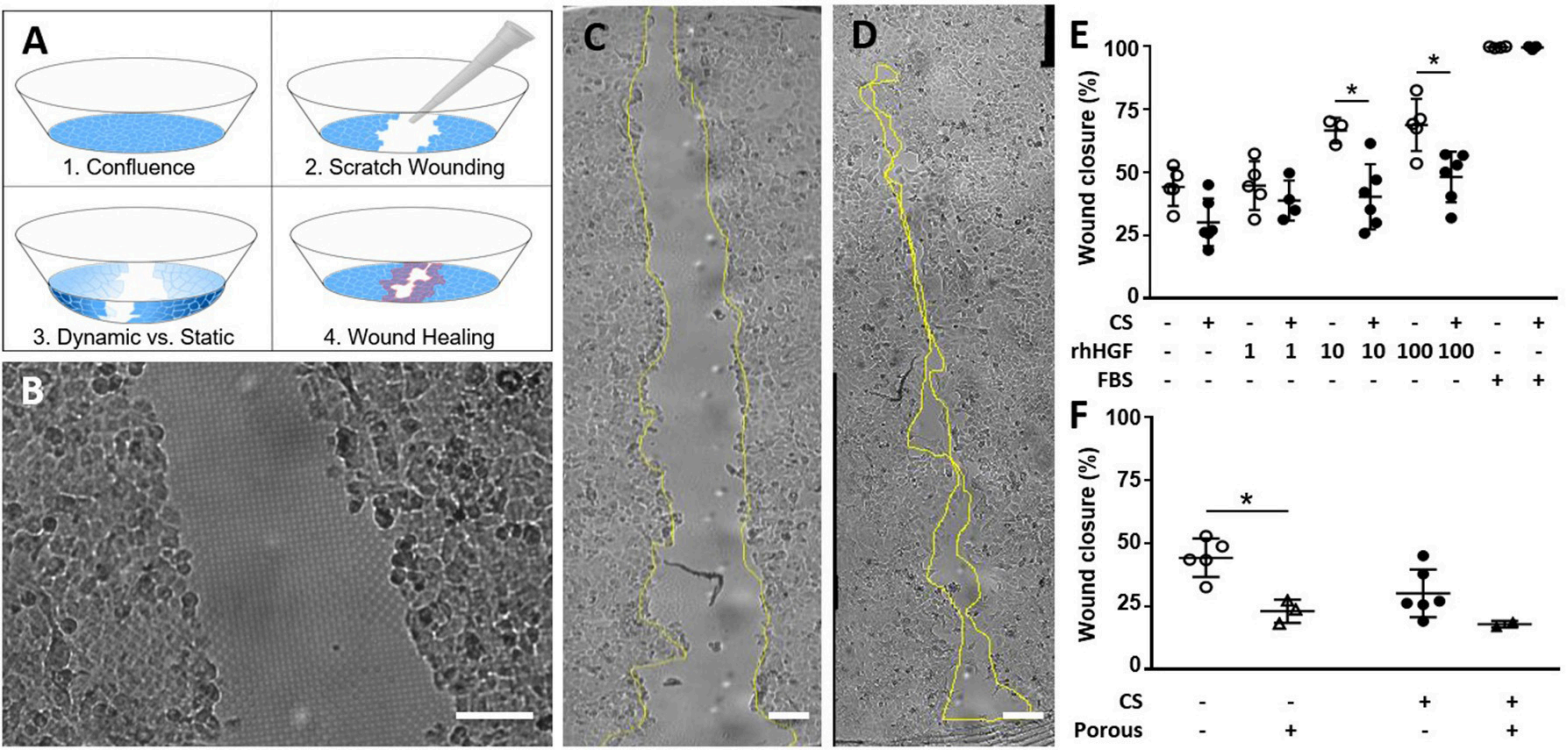
**(A)** Scheme of the scratch wound assay. The wound is created by scratching the cell-covered surface with a standard pipette tip. **(B)** Micrograph of wounded A549 cells on a porous membrane. **(C,D)** Mosaic image of an entire wound immediately after wounding **(C)** and 24 h later **(D)**. **(E)** Wound closure on non-porous membranes, with or without cyclic stretch (CS, 10% linear, 0.2 Hz), recombinant human hepatic growth factor (rhHGF, in ng/mL), or 10% fetal bovine serum (FBS). Wound closure is promoted in a dose-dependent fashion by rhHGF, and impaired when CS is applied. **(F)** Wound closure is impaired when cells are cultured under CS or on a porous membrane. Scale bars: 100 μm.

The epithelial cell monolayer was scratched by dragging a 10 μl pipette tip across the thin membrane (Figures 2A–C). The cells were washed once with starvation medium to remove cell debris. The wounded monolayers were cultured for 24 h in starvation medium with or without recombinant human hepatic growth factor (rhHGF, 1, 10, or 100 ng/ml, R&D Systems, cat#: 294-HG-05). In addition, cells were cultured either under static (no stretch) or dynamic conditions (with three-dimensional stretch corresponding to 10% linear strain at 0.2 Hz).

### Wound-Healing Analysis

Microscopic images of the entire wounded area were taken immediately after wounding and 24 h later. Images taken before wounding served as proof of confluence. The MosaicJ (National Center for Microscopy and Imaging Research: ImageJ Mosaic Plug-ins, RRID:SCR_001935) plug-in for Fiji (RRID:SCR_002285) was used to stitch images together. The wounded area was manually outlined based on images acquired immediately after wounding and 24 h later. Only wounds with areas between 1.2 and 2.5 mm^2^ were analyzed to ensure that data would be comparable. Areas larger than 2.5 mm^2^ (one third of the growth area) were excluded to ensure that sufficient cells were present to repopulate the wounded area. Areas smaller than 1.2 mm^2^ were excluded to ensure that the wound was not completely closed after 24 h.

### Immunofluorescence Microscopy

Cells were immunostained for tight junctions using goat anti–ZO-1 antibody (Abcam, cat#: ab99462) after fixation with 4% paraformaldehyde (PFA, Sigma-Aldrich) and blocking with 2% FBS in PBS. Hoechst-33342 (Molecular Probes, cat#: H3570) was used to stain nuclei. Microscopic images were acquired using a Zeiss Axioplan microscope with a 20 × objective.

### Scanning Electron Microscopy

Cells were fixed for 24 h in 2.5% glutaraldehyde (Sigma-Aldrich) in 10 mM HEPES buffer (Sigma-Aldrich). The cells were dehydrated using a sequence of ethanol concentrations (50–100%). Before images were acquired, the samples were dried using a critical point dryer (Baltec) and sputtered with gold.

### Statistical Analysis

GraphPad prism (RRID:SCR_002798) was used for statistical analyses. All data are shown as means ± standard deviation (SD). A two-way ANOVA with Tukey correction for multiple comparison was used to determine statistical significance.

## Results

Lung alveolar epithelial cells were successfully cultured on the ultra-thin PDMS membrane. Figure 1D shows a confluent layer of cells stained with DAPI (nuclei) and anti–ZO-1 antibody (tight junctions). Cells adhered to the membrane with their filopodia spreading on the porous (Figure 1F) or non-porous silicone membrane (Figure 1E). The elastic membrane resisted the wound creation, where the cell layer was scratched with a pipette tip, and did not tear, despite being only 3.5 μm thick (Figures 2A–C). In medium containing 10% FBS, wound closure was complete after 24 h under both static (99.7%, *n* = 4) and dynamic (99.9%, *n* = 3) conditions (Figure 2E). Because FBS contains a plethora of growth factors, a starvation medium without FBS was used to slow down the wound closure and to measure the effect of HGF alone. In starvation medium, alveolar epithelial wound closure was faster under static than dynamic conditions (44.2 ± 7.6% with no stretching, 30.1 ± 9.5% with cyclic stretching, *n* = 5 and *n* = 6). rhHGF exerted a dose-dependent positive effect on wound closure, irrespective of culture conditions (static or dynamic). In 10 ng/ml rhHGF, wound healing was markedly accelerated under static conditions (44.2 to 66.6%, *n* = 3), and 100 ng/ml rhHGF further increased the wound-healing rate, albeit only slightly (*n* = 5). Under cyclic stress, a similar trend could be observed at 1 ng/ml rhHGF (*n* = 4). At 100 ng/ml rhHGF, wound closure improved from 30.1 to 38.8% (*n* = 6), almost reaching the degree of initial wound closure observed under static conditions.

When the cells were cultured on a porous membrane, wound healing was also significantly slowed relative to a non-porous membrane under static conditions (Figure 2F). With the addition of cyclic stretching, wound healing was delayed further, but not significantly compared to a non-porous membrane (*n* = 3 without stretch, *n* = 2 with stretch).

## Discussion

Wound-healing assays are widely used in drug discovery and in fundamental investigation of the pathogenesis of wound healing and of scar formation. Several organs, including the skin and lung, are affected by diseases whose onset is related to wounding. In the lung, IPF and acute lung injuries are severe diseases for which the wound-healing mechanisms are crucial in the development of the disease, but the cellular and molecular mechanisms are not completely understood. Unfortunately, *in vivo* models are of limited utility for investigating wound repair in the lung, in part because it is not possible to induce single small wounds and observe their repair over time. However, standard *in vitro* techniques are also limited because they poorly reproduce the *in vivo* milieu; instead, such systems often consist of a monolayer of cells cultured on a hard substrate and submerged in a static medium. The microenvironment of the lung, by contrast, undergoes constant mechanical stimulus induced by breathing motions. In addition, in a healthy lung, epithelial cells are exposed to air, and the tissue contains many other cell types. Recent advances in microengineering and cell biology have made it possible to develop advanced systems that mimic these properties (Huh et al., 2010; Stucki et al., 2015, 2018). We therefore assume that this *in vitro* model of epithelial wound repair including mechanical forces represents the *in vivo* situation at a higher level than conventional *in vitro* model systems.

Here, we performed a proof-of-concept study of a wound-healing assay on a lung-on-a-chip. Applying cyclic mechanical stretch reduced the wound-healing capabilities of alveolar cells over 24 h, consistent with studies of human bronchial cells and alveolar epithelial cells in rodents and humans (Desai et al., 2010; Crosby et al., 2011; Ito et al., 2014). The impaired wound closure was partially restored by the addition of rhHGF. This effect was concentration-dependent in all conditions, confirming earlier results (Felder et al., 2012, 2014; Ito et al., 2014). The mechanism underlying the impaired wound repair upon stretch is not completely understood. Desai et al. showed that cyclic stretch corresponding to 20% linear strain at 0.5 Hz inhibited phosphatidylinositol 3-kinase (PI3K) in bronchial epithelial cells (Desai et al., 2010). PI3K is typically activated in wound healing. However, Ito et al. did not observe the same downregulation in PI3K when alveolar cells were subjected to only 10% linear strain at 0.1 Hz (Ito et al., 2014). These discrepancies suggest that different pathways may be involved in wound healing depending on the stretch magnitude and frequency. However, other players, such as stiffness, which can importantly affect the strain level, are involved in IPF remodeling (Selman and Pardo, 2014). In the present lung-on-a-chip, the stiffness of the membrane may be modified in the future to mimic this aspect.

Furthermore, we observed reduced wound healing on a porous membrane (3 μm pores, 800,000 pores/cm^2^) relative to a non-porous membrane, regardless of cyclic stretch. This can be partially explained by the fact that, on porous membranes, there is no continuous cell support. The interaction between cells and the extracellular matrix (ECM) plays an important role during growth, development and repair (Rosso et al., 2004). The absence of this ECM–cell interaction where the pores are located may slow down cell migration. Furthermore, A549 cells are capable of transmigrating through the 3 μm pores to the other side of the membrane and are thus not involved in the wound closure.

The semi-open design of the lung-on-a-chip provides an easy access to the apical cell culture well. Consequently, the cell layer can simply be wounded with a pipette tip, and wound healing can be observed during cyclic mechanical stretching. To the best of our knowledge this is the first report of a wound-healing assay performed on an ultra-thin, porous, and stretchable membrane. This system enables the development of more complex assays that can so far not be reproduced.

Preliminary results culturing epithelial cells with fibroblasts (see Supplementary Materials) show that wound-healing of a co-culture model can be investigated with this device. This is of great importance to study the pathophysiology of interstitial lung diseases. By further adding immune cells and/or endothelial cells, other biological questions may be investigated. In addition to the cyclic stress, other aspects of the unique lung alveolar environment can be emulated. Notably, the air-liquid interface, which all healthy pulmonary epithelial cells are exposed to. In such configuration, the epithelial layer receives its nutrients via the basolateral side of the membrane (through endothelial cells) while being exposed to air on the apical side. The reproduction of these key environmental aspects may help to fully elucidate the complex interplay between mechanical strain and deficient alveolar wound repair that underlies IPF and other diseases. By culturing diseased cells from patients, our hope is to be able to develop cell-based assays for precision medicine.

## Author Contributions

OG and TG had the original idea to investigate microinjuries on a chip exposed to mechanical stimulation. MF, BS, and OG planned the microinjury study. BT, MF, and SB carried out the experiments involving the microinjuries. AS performed the immunostaining. JS prepared the samples for the SEM experiments and SEM imaging. AS, OG, MF, and BT wrote the manuscript. All authors reviewed the manuscript.

### Conflict of Interest Statement

OG, TG, and JS are shareholders of the start-up AlveoliX AG, which aims at bringing to market a lung-on-a-chip system based on the model described in this article. The remaining authors declare that the research was conducted in the absence of any commercial or financial relationships that could be construed as a potential conflict of interest.
