# Supplementary material for: Impaired Wound Healing of Alveolar Lung Epithelial Cells in a Breathing Lung-On-A-Chip
Source: Front Bioeng Biotechnol. 2019 Jan 22;7:3. doi: 10.3389/fbioe.2019.00003 (PMC6360510; doi:10.3389/fbioe.2019.00003)
Supplement: Supplementary file 1 [file Data_Sheet_1.pdf]

Supplementary Material

Impaired Wound Healing of Alveolar Lung Epithelial Cells in a Breathing Lung-on-a-chip

Marcel Felder[*], Bettina Trueeb[*], Andreas Oliver Stucki[*], Sarah Borcard, Janick Daniel Stucki, Bruno Schnyder, Thomas Geiser, Olivier Thierry Guenat[#]

***** These Authors contributed equally to the manuscript
# Correspondence: Olivier Guenat: olivier.guenat@artorg.unibe.ch

1 Co-Culture of Epithelial cells and Fibroblasts on Chip

1.1 Introduction

Idiopathic pulmonary fibrosis (IPF) is the most common form of interstitial lung diseases (Coultas et al., 1994), which are characterized by pronounced tissue remodelling with marked thickening of the interstitial tissue at the gas-exchanging airways (Visscher & Myers, 2006). IPF has long been thought to be due to chronic low-grade inflammation in the airways. Failure of anti-inflammatory therapies in fibrosis patients, however, has challenged this hypothesis and led to a paradigm shift in pulmonary research. Currently, fibrosis is supposed to be the consequence of repetitive epithelial injuries, followed by aberrant wound healing processes (King et al., 2011).

Injuries to the vulnerable alveolar epithelium initiate a cascade of signaling events that lead to wound clot formation in the alveolus, basal membrane disruption and fibroblast proliferation, infiltration and differentiation towards contractile myofibroblasts. The infiltrated and residual myo-/fibroblasts then secrete extra-cellular matrix (ECM) components, mainly collagens, that set the stage for efficient re-population of the denuded basal membrane. In healthy lungs, the alveolar epithelium is repopulated by proliferating type II alveolar epithelial cells that spread, migrate to the initial wound area and differentiate into type I AEC (Evans et al., 1973; Fehrenbach, 2001). Upon complete restoration of the epithelial barrier, the provisional wound clot is then resolved and the alveolus returns to full-function again (Beers & Morrisey, 2011; Crosby & Waters, 2010). In IPF lungs, however, the normal wound repair is deregulated, and fibroblasts are continuously recruited to the alveolar space. Consequently, fibroblasts accumulate in the sub-epithelial region, form local clusters (fibroblast foci) and secrete excessive amounts of ECM that critically impair the normal gas exchange. In the attempt to close the wounds in the persistently apoptotic epithelium, type II alveolar epithelial cells continuously proliferate and become hyperplastic (Bachofen & Weibel, 1974). The inefficiency of the alveolar wound healing process leads to sustained bi-directional repair signals that further fuel the vicious circle underlying fibrosis. Hyperplastic epithelium and fibroblast foci are typical hallmarks of fibrotic lung tissue and were shown to be in close proximity to each other(Kuhn et al., 1989; Selman & Pardo, 2002). In conclusion, the normal repair mechanisms of the respiratory epithelium seems to be overwhelmed in IPF, which leads to massive fibroblast infiltration and profound airway remodeling(Selman et al., 2001; Selman & Pardo, 2002).

Moeller et al. reported that none of the approximately 250 therapeutic agents, which were successfully tested in animal models of pulmonary fibrosis, has gained clinical acceptance (Moeller et al., 2008). The relevance of these animal models has therefore been questioned and attempts are undertaken, to overcome some of these limitations with advanced in vitro models like the here presented lung-on-a-chip.

1.2 Methods

1.2.1 Co-culturing of alveolar epithelium and fibroblasts

The porous membranes were coated with a mixture of 6.66 μg/cm^2 human fibronectin (Corning, Cat#: 354008) and 18.18 μg/cm^2 collagen 1 (Sigma-Aldrich, cat# C4243) for 24 h prior to cell seeding. A549 lung alveolar epithelial-like cells (p22, ATCC cat#: CCL-185, RRID:CVCL_0023) were cultured in RPMI 1640 culture medium with GlutaMAX™ (Gibco/Life Technologies, cat#: 61870-010) supplemented with 10% fetal bovine serum (FBS, Gibco/Life Technologies, cat#: 10270-1 06) and 1% penicillin–streptomycin (Gibco Life Technologies, cat#: 15140-122) at 37°C and 5% CO_2. The cells were labeled with a red fluorescent membrane dye (PKH26, PTI Research, Inc.) prior to seeding 50,000 cells/cm^2 on the apical side of the alveolar membrane. After 24 h, normal human lung fibroblasts (NHLF, Lonza, cat#: CC-2512) were seeded on the basal side of the membrane at a concentration of 10,000 cells/cm^2 (Figure S1). 4 h after the seeding of the fibroblasts, the medium was changed to serum-free medium for another 20 h.

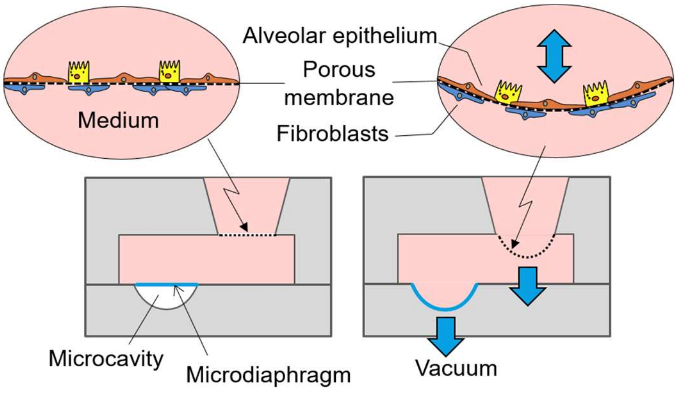

Supplementary Figure S1: Schematic of the lung-on-a-chip with a co-culture of alveolar epithelium and fibroblasts.

1.2.2 Scratch wounding

The epithelial cell monolayer was scratched by dragging a 10 μl pipette tip across the thin membrane (Figure 2A-C). The cells were washed once with starvation medium to remove cell debris. The wounded monolayers were cultured for 24 h in serum-free medium. Cells were cultured either under static (no stretch) or dynamic conditions (with three-dimensional stretch corresponding to 8% linear strain at 0.2 Hz).

1.3 Results and Discussion

A549 alveolar epithelial-like cells were cultured for 24 h before normal human lung fibroblasts were seeded on the other side of the porous membrane (Figure S1). The co-culture could be successfully established and maintained even in serum-free medium (Figure S2A). Before wounding the A549

cells formed a nice and confluent cell layer regardless of whether fibroblasts were cultured on the basolateral side of the porous membrane or not (Figure S2C). Furthermore, wounds of a few hundred micrometers in width were successfully created using a 10µl pipette tip (Figure S2B). Fluorescence imaging could be performed at several timepoints after the wounding of the cells to monitor the wound healing progression. This proof-of-concept experiment shows, that it is possible to co-culture lung fibroblast with alveolar-like epithelial cells and to create a wound in the epithelial layer.

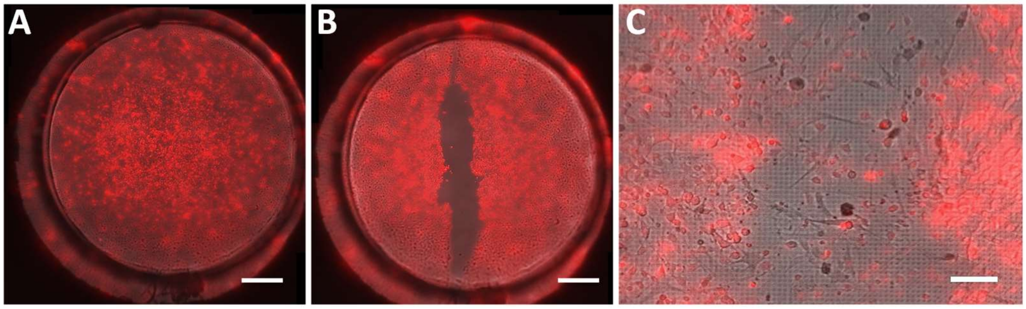

Supplementary Figure S2: A+B Micrographs of the confluent alveolar epithelial layer before and immediately after wounding with a 10µm pipette tip. Alveolar epithelial cells were stained using a live cell membrane dye in red. Scale bar: 0.5 mm. **C** Closer view on the wounded co-culture of A549 (stained in red) and normal human lung fibroblasts (elongated cells) growing on the other side of the porous membrane. Scalebar: 100 µm.

1.4 Conclusion

Preliminary tests of an alveolar epithelial/fibroblasts co-culture on an ultra-thin, porous and flexible membrane were successful. In addition, the epithelial layer could be wounded by a scratch assay and imagined. Further experiments will focus on the complex interplay between both cell types during wound-healing in a dynamic environment.

1.5 References

Bachofen, M., & Weibel, E. R. (1974). Basic pattern of tissue repair in human lungs following unspecific injury. *CHEST*, *65*, 14–19. https://doi.org/10.1378/chest.65.4_Supplement.14S

Beers, M. F., & Morrisey, E. E. (2011). The three R's of lung health and disease: Repair, remodeling, and regeneration. *Journal of Clinical Investigation*. https://doi.org/10.1172/JCI45961

Coultas, D. B., Zumwalt, R. E., Black, W. C., & Sobonya, R. E. (1994). The epidemiology of interstitial lung diseases. *American Journal of Respiratory and Critical Care Medicine*, *150*, 967–972. https://doi.org/10.1164/ajrccm.150.4.7921471

Crosby, L. M., & Waters, C. M. (2010). Epithelial repair mechanisms in the lung. *American Journal of Physiology. Lung Cellular and Molecular Physiology*, *298*(6), L715-31. https://doi.org/10.1152/ajplung.00361.2009

Evans, M. J., Cabral, L. J., Stephens, R. J., & Freeman, G. (1973). Renewal of alveolar epithelium in the rat following exposure to NO2. *The American Journal of Pathology*, *70*, 175–198.

Fehrenbach, H. (2001). Alveolar epithelial type II cell: defender of the alveolus revisited. *Respiratory Research*, *2*(1), 33–46.

Guenat, O. T., & Berthiaume, F. (2018). Incorporating mechanical strain in organs-on-a-chip: Lung

and skin. *Biomicrofluidics*, *12*(4). https://doi.org/10.1063/1.5024895

King, T. E., Pardo, A., & Selman, M. (2011). Idiopathic pulmonary fibrosis. *Lancet*, *378*(9807), 1949–1961. https://doi.org/10.1016/S0140-6736(11)60052-4

Kuhn, C., Boldt, J., King, T. E., Crouch, E., Vartio, T., & McDonald, J. a. (1989). An immunohistochemical study of architectural remodeling and connective tissue synthesis in pulmonary fibrosis. *The American Review of Respiratory Disease*, *140*, 1693–1703. https://doi.org/10.1164/ajrccm/140.6.1693

Moeller, A., Ask, K., Warburton, D., Gauldie, J., & Kolb, M. (2008). The Bleomycin animal model: A useful tool to investigate treatment options for idiopathic pulmonary fibrosis? *The International Journal of Biochemistry & Cell Biology*, *40*(3), 362–382. https://doi.org/10.1016/j.biocel.2007.08.011

Selman, M., King, T. E., & Pardo, A. (2001). Idiopathic pulmonary fibrosis: prevailing and evolving hypotheses about its pathogenesis and implications for therapy. *Annals of Internal Medicine*, *134*(2), 136–151.

Selman, M., & Pardo, A. (2002). Idiopathic pulmonary fibrosis : An epithelial / fibroblastic cross-talk disorder. *Respiratory Research*, *8*, 1–8.

Visscher, D. W., & Myers, J. L. (2006). Histologic spectrum of idiopathic interstitial pneumonias. *Proceedings of the American Thoracic Society*, *3*(8), 322–329. https://doi.org/10.1513/pats.200602-019TK
